# Topics of Public Interest

**Published:** 1914-07

**Authors:** 


### TOPICS OF PUBLIC INTEREST

---

**Long Island College Hospital, Brooklyn,** has undergone complete organization in order to meet the modern requirements of teaching medicine. It has instituted a five year course to take effect in September of this year, and has arranged to add over twenty fulltime members to its faculty and every department has been increased. The junior year will be given over to dispensary work and didactic medicine and surgery, and the senior year will be devoted entirely to bedside work in the hospital owned by the college, which, with the new addition, will give the institution 560 beds and make it one of the largest in Greater New York.

The following gentlemen will occupy the new positions on the faculty:

Dr. Archibald Murray, Professor of Pathology.
Dr. William Lintz, Professor of Bacteriology.
Dr. John C. Cardwell, Professor of Physiology and Pharmacology.
Dr. Matthew Steel, Professor of Chemistry.
Dr. William Francis Campbell, Professor of Surgery.
Dr. Joshua M. Van Cott, Professor of Clinical Medicine.
Dr. William B. Brinsmade, Professor of Clinical Surgery.
Dr. E. H. Bartley, Professor of Pediatrics.

---

**The Buffalo General Hospital** graduated a class of 22 in June.

---

**Dental Dispensaries,** for Buffalo have been opened at 1067 Grant street and William and Stanton streets.

---

**Electric Disinfection of Water.** The first plant of this kind in America is being installed at Niagara Falls. Ultra-violet rays will be generated by electric lights through lenses containing mercury.

---

**Blanks for Heroin, Morphine, Codeine, etc.** The Martin H. Smith Co. call attention to the fact that such blanks must be obtained by physicians, in accordance with Chapter 363 of the Health Laws of New York. Application should be made to the Secretary of the State Board of Health at Albany.

---

**American Doctor Studies Typhus.** Dr. Clarence D. Ussher, has received public thanks from the vali of the Turkish vilayet of Van for his ''humane zeal and care for the sick'' in the recent epidemic of typhus among the Turkish soldiers quartered in the city of Van. Dr. Ussher is in charge of the American Board's hospital in Van. He is a graduate of Kansas Medical College and has been in Turkey since 1898.

Van, the city, is the center of the Turkish province of the same name, away up in the northeastern corner of Asiatic Turkey. On its eastern border the vilayet joins Persia while on the north it is very near the Russian Caucasus. The city is on the shore of Lake Van, a large sheet of water whose surface is 5,500 feet above tide level. The city is said to have been built by Semiramis and strange and interesting inscriptions and carvings are found in and about it.

Since the American Board began work there, the mission has been through war, famine, pestilence and massacre and the epidemic of last winter will take its place in the history of calamities which Americans have helped the people to fight.

The Turkish military authorities were not insistent on cleanliness and sanitation and over 2,500 soldiers have died of the disease. Dr. Ussher was active wherever he was permitted to serve but had not access to the barracks or to the military hospital. In the Board's hospital, however, and in going about among the sick in the camp and in the city he made careful study of the disease and says:

"We have proved very conclusively in our hospital that the only means of infection is vermin. Our nurses have been thoroughly exposed to every form of contagion from the breath, desquamation, discharges, constant association day and night, and all this in an over-tired condition, and not one of them contracted the disease. The typhus patients have been put in the same ward with surgical, pneumonia, dysentery and even confinement cases, and not a single patient has become infected in the hospital. One of our nurses subjected himself to infected body lice, and promptly contracted the disease (incubation I think five days). I personally removed the lice from his body. We have become so sure of the mode of infection that, being compelled by the lack of bedding, we put patients with other trouble in the beds which had been occupied by typhus patients. We made no further change than clean sheets and pillow covers; and though in several cases the limit of incubation has passed twice over there has not been a single infection."

Two million people and only one doctor. Seven doctors, each with a territory holding two million souls. These are the facts of the medical situation in the part of China around the American Board's Shansi Mission. In addition to the surgical cases which are brought from many miles distant to Dr. Percy T. Watson at the Board's Dispensary in Fenchow-fu, the prefectural city which is the Shansi Mission's headquarters, there are many out-stations where patients are cared for in school rooms, in inn yards or wherever the doctor can find a vacant room on his tours, while seven or eight opium refuges are managed from Fenchow-fu.

An opium refuge has just been reopened by American Board workers at Yulin-fu, a city of Shensi, the province next west of Shansi. Yulin-fu is in the shadow of the Great Wall and is the gateway of Mongolia from all western China. No missionary work has been done there since the Boxer uprising in 1900 when the opium refuge then in operation was destroyed. A medical station there would do untold good. But only seven physicians to fourteen million people. Where are the young American doctors having a passion for service and in search of a practice?

American Board of Commissioners for Foreign Missions,
Congregational House, 14 Beacon street, Boston, Mass.

---

**Too Much Morality.** Last month, a physician with his chauffeur, in a one-seated auto, accomodated a young lady with a ride. She sat, as convenience and safety required, on the doctor's knee. The doctor's wife brought action on the grounds of an offense against public decency. The case was dismissed. This is a sensible decision. There are probably very few men using one-seated vehicles of any kind who have not done the same thing. We have even seen a man sitting on a woman's lap in a street car. It is wise not to emphasize the sexual element by prudishness.

A jury recently refused to find a youth guilty under the Mann act. There was, apparently, no doubt of his having gone on a trip, outside of the state, with a young woman and the Mann act, ostensibly designed to oppose white slavery, has been construed to be a censor of morals without regard to the elements of commercialism, coercion or undue suasion. We do not wish to be understood as in any sense upholding immoral practices but we do not believe that any country can be made good by law and we consider it a dangerous precedent to extend criminal legislation beyond the range of direct damage to person and property. In the case mentioned, the evidence showed clearly that, if anything, the man was the injured party, as he claimed to have been made a heroin victim by the woman. If the Mann act is to be construed as a general censor of morals, let it apply equally to both sexes, with due regard for circumstances.

---

**The Psychics of Female Suffrage.** There are two points in this connexion that seem to deserve serious medical inquiry. What process of cerebration implies the demonstration of absolute unfitness for citizenship as an argument for obtaining it? Why, in similar countries, of the same language, with not much difference in race of the active parties, and with nearly the same legal and social conditions, England and the U. S., do we have such marked contrasts of dastardly violence and of rational and temperate argument with, at most, the use of a few rather spectacular methods, such as "hikes." Can any one imagine the women who participated in the recent suffrage parade in Buffalo, smashing antiquities, violating the sanctity of churches, horsewhipping officials, and setting bombs?

---

**Guarantees Under the Pure Food and Drug Act**: June 30, 1906. The Dept. of Agriculture has at last taken definite action in regard to the silly method of attempting to secure pure foods and drugs. As might have been anticipated by anyone with the slightest acquaintance with business methods, this law has been exploited to imply a government guarantee of privately manufactured products and while rulings were made to prevent this natural tendency, the fact remained that any average person not informed to the contrary, would so understand the legally required addition to the label. On the other hand, manufacturers and others who naturally understood that provision would be made, as rapidly as possible for systematic examinations of claims of manufacturers, have been disappointed. Many manufacturers were not only willing but anxious to submit samples for analysis; in some cases to establish merit in which they felt full confidence, in others to prevent any possible violation of either the spirit or letter of the law in regard to doubtful points. That the government could not immediately attend to all these cases was obvious; that it should have no intention of doing so after having passed the law establishing general principles, was a curious inconsistency. 58,816 serial numbers have been assigned up to the present. No more numbers will be assigned but guaranties are to be attached to invoices. After May 1, 1915, the same system will apply to those now in possession of serial numbers, and such numbers and guaranties are to be omitted from labels. This is a wise move but it is incomplete. There should be, not a haphazard indictment of violators of the law but, having established the general principle that drug and food preparations must be pure and honest, there should be a systematic method of controlling them and of holding for reference, analyses. It should be distinctly understood that these criticisms are not directed against the highly efficient Dept. of Agriculture, which is limited in its scope by its appropriation, but to the legislative lack of foresight or even hindsight, exemplified in the law itself.

---

**Advertising Principles.** The Monroe Co. Medical Society has approved the following principles adopted by a joint committee of representatives of the Ad Club, of the Chamber of Commerce, of the Rochester Ministerial Union, of the Rochester Dental Society, of the Rochester Optometric Society, of the Monroe County Medical Society, of the Monroe County Homeopathic Medical Society and some of the newspapers.

### PRINCIPLES AND PROHIBITIONS GOVERNING ADVERTISING.

### Principles.

1. That advertising is objectionable which adversely affects the public health, morals or pocketbooks.

2. Anyone who accepts advertising for publication or display, in any medium he controls, is under moral obligation to protect the readers of, and legitimate advertisers in, such medium, against objectionable advertising.

3. Anyone offering advertising for publication or display, is equally responsible for the honesty, truthfulness and reliability of such advertising.

To vitalize the spirit of the principles above set forth, we adopt the following specific prohibitions:

### Prohibitions.

I. Absolute and total, against

1. Objectionable medical advertisements.

2. Suggestive or indecent advertisements.

II. Partial or provisional, against

1. Offers of something for nothing or apparent mercantile impossibility.

2. Doubtful financial, mining, real estate, or other business propositions.

3. Any advertisment which fairly raises a suspicion of fraud.

With these principles we are in hearty accord . During the last three years we have refused about $600 worth of advertising designated as II 2. It should be understood that we cannot personally guarantee the profitability of individual investments nor can we inforce our personal conviction that the small investor should keep to real estate and mortgages upon it, near enough so that he can give it personal supervision. But, in every instance in which such an advertisement has been tendered, we have taken every precaution in the interests of our readers. It must also be considered that there is some difficulty, especially in a medical journal, in deciding what advertisements are objectionable. One of our contemporaries which is very strong on ethics has published three that we consider highly objectionable and, we are informed by the firms interested, has decided similarly against two others, after giving the manufacturers the opportunity to increase their advertising space. We have in mind an advertisement, considered unethical, which, on examination, showed merely bad taste in its lay-out and which conformed to every possible test of ethics. Again ethics is sometimes construed to mean that one may advertise a powerful drug and not a mild one; that a journal may advertise a firm which carries a hundred stock formulae, not one that concentrates on one or a few. In a recent case, the admission of an advertisement was considered unethical because the instrument advertised was unscientific and merely practical and approximate. It is obvious that a grading of this sort is out of the question, although we have excluded one advertisement because of the poor quality and inaccuracy of apparatus. It is obvious, on reflection, that a medical journal cannot apply the same rules to a merchant of ordinary wares, as to a manufacturer of drugs, in regard to insistance on lay advertising. There are a great many groups of advertisements; logically appearing in medical journals but applying to articles in common use as domestic remedies, beverages, use in cleansing and disinfecting, etc. In such cases as this, we endeavor to formulate a fair standard for each class and to apply it to all advertising of the class. For instance, beverages can not be excluded from lay advertising although, personally, we would go as far as to claim that, under ideal conditions, alcoholic beverages should be used only as drugs. This ideal is at present utterly impracticable. It can be achieved only by admitting alcoholic beverages to the class of medical advertising and by gradually restricting their use and advertisement in these directions. This being the case, no one article of this class can be singled out for opprobrium because of lay use and lay advertising. It has been ruled that the name of a drug should not suggest its therapeutic use. We recognize the force of the arguments but must admit that the suggestion of the use of a drug is both convenient and even a factor of safety. We do not see how the rule can be enforced as a principle of ethics, so long as emetine, morphine and pilula cathartica composita and numerous other official names remain in the Pharmacopoeia. Neither do we consider that this rule or any other should be applied post facto, until this country removes its constitutional objection to post fact legislation and this, we hope, will never occur. In short, the term "objectionable" must be applied in a reasonable and fair way, subject to differences of individual opinion. There is nothing quite so potent as the lime light of publicity to enable close scrutiny of anything that is doubtful. Close attention to advertisements and discussion of such points as may determine their ethical status, is the best means of determining efficient and practical control of objectionable advertising.

---

**The Robert W. Long Hospital** was dedicated June 15, exercises being held in the Chamber of the House of Representatives at Indianapolis.

---

**The Open Air Camp of Buffalo,** has begun its seventh season. It is located on Grider street near Kensington avenue. Resident physicians and nurses are provided but cases may remain under the care of the physician sending them. Children will not be admitted this season as provision has been made for them at the J. N. Adam Memorial Hospital at Perrysburg. Applications should be made at the Tuberculosis Dispensary, 175 East Swan street.

---

**The Samuel D. Gross Prize of $1,500** will be awarded on essays received in competition up to January 1, 1915. Address the Trustees, 19 S. 22, Philadelphia, for detailed information.

---

**Ancient Drug Store Loafers.** In an apothecary's shop in Pompeii, has been found the following inscription: "Otiosus non est Locus. Discede morator." Not a resting place. Idler get out.

---

**Imitations and Substitutes.** The Purdue Frederick Co. have asked us to refer to this matter—which needs no argument—not only in their own interests but as a broad issue affecting the medical profession and the people.

---

**Prison Epidemic of Scarlet Fever.** Over 1,000 cases, most of them mild, have occurred in the last few weeks at Auburn. Uninfected men whose terms are nearly expired, will be discharged after a week's quarantine.

---

**Alleged Leper at Large.** John R. Early, whose case has attracted so much attention in the last five years, the diagnosis being questioned by some, escaped from the Diamond Head Quarantine Station near Port Townsend, Wash., May 18, was discovered at a fashionable residence hotel in Washington, D. C., June 2.

---

**Small-pox in Mexico.** This disease was formerly a scourge of military camps. It has recently broken out and has shown a high death rate in the Mexican Constitutionalist forces at Suliacan, the capital of Sinaloa.

**Public Health Nurse's Service as Prize.** Webster Grange No. 436, near Rochester sold the largest number of Christmas Tuberculosis seals in last year's contest and has been awarded the services of a nurse, Miss Elizabeth Hanson, as the prize. Lewiston Grange, Niagara Co., won the second prize. Miss Hanson will devote two months to work in Webster and will then proceed to Lewiston.

